# Cardiac Anomaly: Reverse Takotsubo Following Gallbladder Surgery

**DOI:** 10.7759/cureus.65297

**Published:** 2024-07-24

**Authors:** Sandres Aodish, Ryan Tam, Modather Grain, Bryan Fiema

**Affiliations:** 1 Surgery, Lake Erie College of Osteopathic Medicine, Erie, USA; 2 Internal Medicine, Lake Erie College of Osteopathic Medicine, Erie, USA; 3 Internal Medicine, Unity Hospital, Rochester, USA; 4 Cardiology, Unity Hospital, Rochester, USA

**Keywords:** laparoscopic cholecystectomy, post-laparoscopic cardiomyopathy, stress induced cardiomyopathy, reverse takotsubo cardiomyopathy, takotsubo cardioyopathy

## Abstract

Takotsubo cardiomyopathy is an acute but often reversible left ventricular dysfunction commonly triggered by emotional stress. There are multiple variants within the general condition; however, reverse Takotsubo cardiomyopathy is a rare variant of stress-induced cardiomyopathy affecting the basilar segment of the left ventricle. This commonly manifests in younger women with clinical presentations similar to acute coronary syndrome. Cases of postoperative reverse Takotsubo cardiomyopathy are limited in the current literature. Hence, we present an 81-year-old female with shortness of breath and chest pain with exertion following a recent laparoscopic cholecystectomy. Based on her symptoms during the presentation, troponin and beta-natriuretic peptide were obtained. Results demonstrated an elevation in both markers, raising concerns for possible acute coronary syndrome (ACS). The patient subsequently underwent a transthoracic echocardiogram (TTE), which demonstrated findings consistent with reverse Takotsubo cardiomyopathy (rTTC). Therefore, we present a unique case of an 81-year-old female presenting with rTTC following laparoscopic cholecystectomy.

## Introduction

Takotsubo cardiomyopathy (TTC), also known as "stress cardiomyopathy" or "broken heart syndrome," is a reversible acute cardiac condition that mimics acute coronary syndrome (ACS) [1,2]. Although TTC mimics ACS, there is generally no evident coronary obstructive disease on angiogram [1]. TTC is characterized by transient cardiac apical akinesis/hypokinesis and basal hyperkinesis on an echocardiogram, which resolves within a few weeks [1,2].

There are four types of TTC currently described in the literature: the typical form with apical ballooning, mid-ventricular form, focal form, and basal form, also known as reverse TTC (rTTC) [2]. rTTC is a rare variant, with an incidence of just 2.2% of all reported Takotsubo cases from the International Takotsubo Registry [1]. This rare variant presents with basal ballooning instead of the typical apical ballooning, which accounts for 80% of all cases [2].

TTC typically presents in older postmenopausal females, whereas rTTC generally occurs in younger females [3,4]. Patients with rTTC usually have a lower ventricular ejection fraction and quicker recovery time than in TTC [3].

## Case presentation

An 81-year-old female with a past medical history significant for chronic obstructive pulmonary disease on two liters of oxygen via nasal cannula, mild nonobstructive coronary artery disease, an ascending aortic aneurysm measuring 5.1 cm, and demand-mediated non-ST elevation myocardial infarction presented to the emergency department with shortness of breath. Her past surgical history was remarkable for a laparoscopic cholecystectomy with the placement of a Jackson-Pratt/Blake drain with closed bulb suction in the gallbladder fossa performed six days before her presentation to the emergency department.

Upon admission, the patient appeared to be a frail, ill-appearing elderly woman with moderate respiratory distress. The patient reported being tired during the period immediately following the surgery. Earlier in the day, before coming to the emergency department, she had the Jackson-Pratt/Blake drain removed at the outpatient surgical follow-up, where she endorsed chills, malaise, and worsening shortness of breath that began four days prior. Additionally, there were reports of occasional chest pain on exertion.

**Figure 1:**
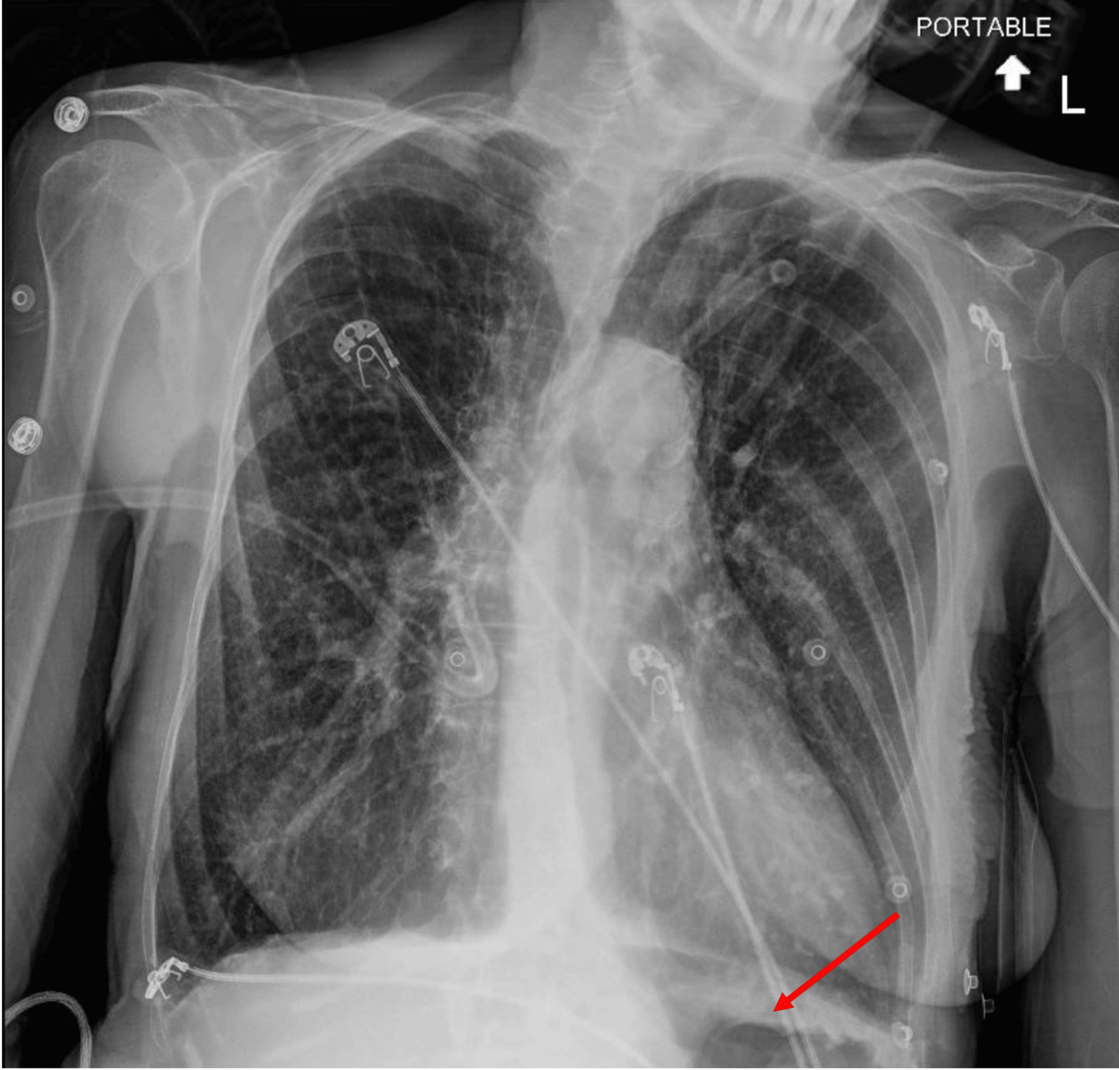
Chest radiograph demonstrating air under left diaphragm (red arrow) and bilateral interstitial opacities.

**Figure 2:**
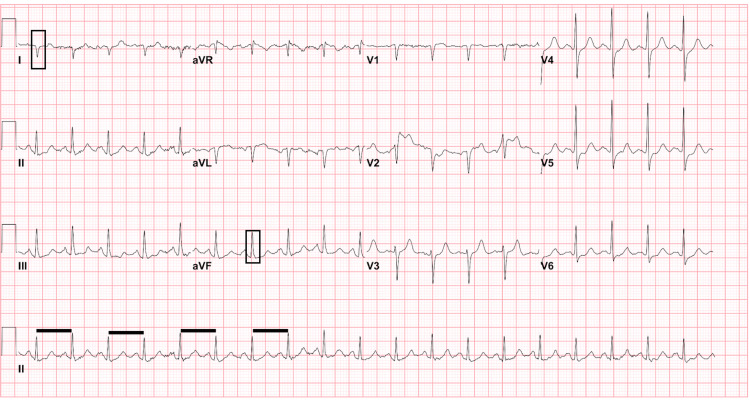
ECG demonstrating sinus tachycardia (decreased R-R interval demonstrated by black lines) with right axis deviation (black boxes in leads I and aVF). Nonspecific ST segment changes without elevation were also present.

As a result, a chest radiograph (Figure 1) was performed, which demonstrated apparent gas under the left diaphragm and interstitial opacities bilaterally. An electrocardiogram (ECG) was obtained which was remarkable for sinus tachycardia and right axis deviation (Figure 2). Serum high sensitivity troponin-I was performed, demonstrating normal levels of 26 pg/mL at initial presentation, followed by elevated levels at 129 pg/mL (reference range: <50 pg/mL) one hour later. Beta natriuretic peptides were also found to be mildly elevated at 114 pg/mL (reference range: <100 pg/mL).

**Figure 3:**
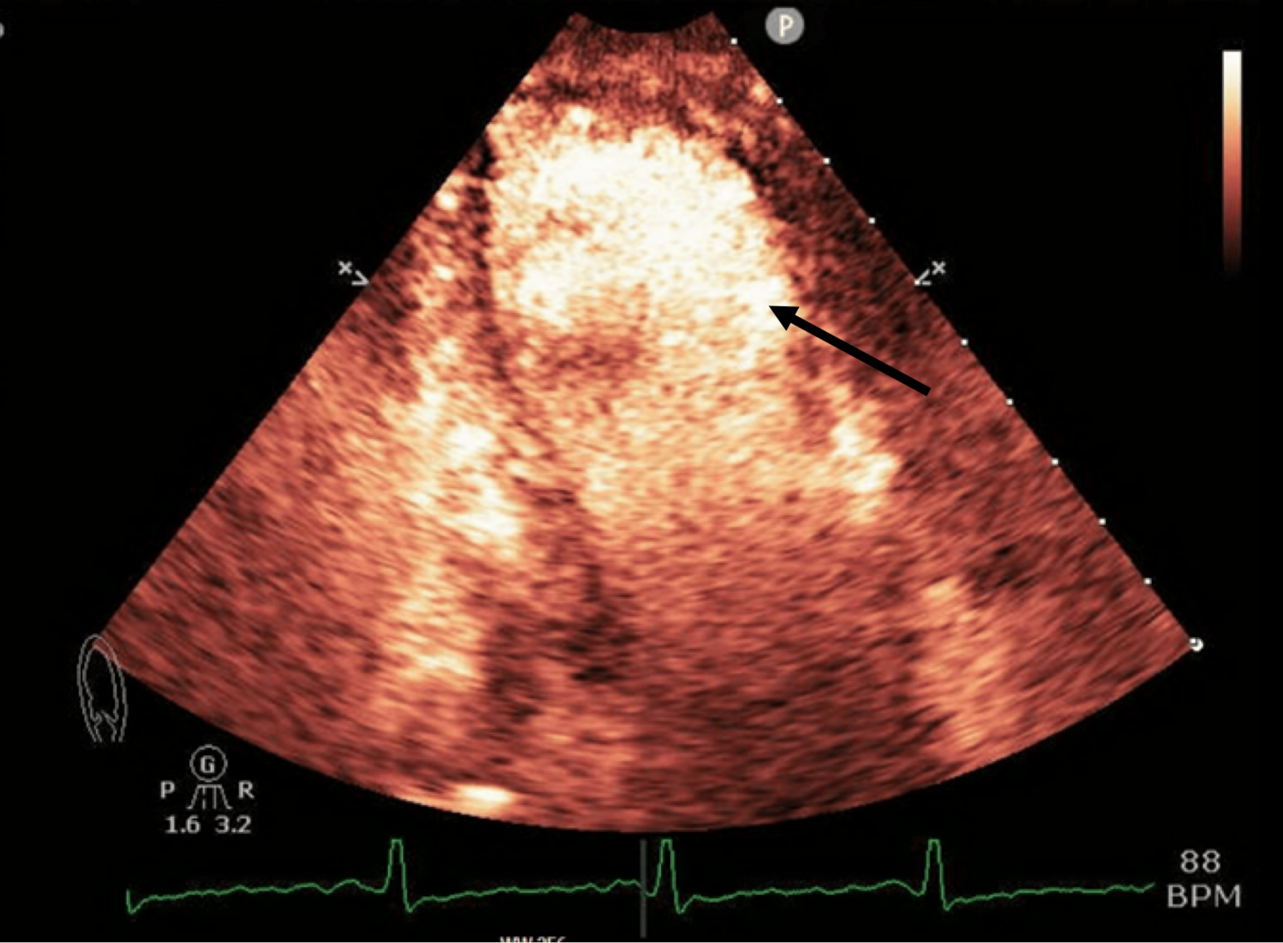
Two-chamber echocardiograph demonstrating ballooning of the basal segment at the end of systole indicated by black arrow.

**Figure 4:**
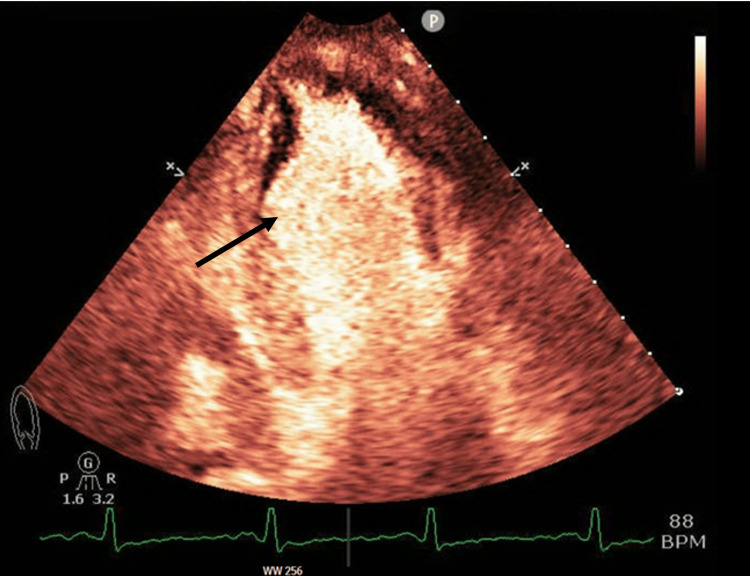
Two-chamber echocardiograph demonstrating ballooning of the basal segment at the end of systole indicated by black arrow.

**Figure 5:**
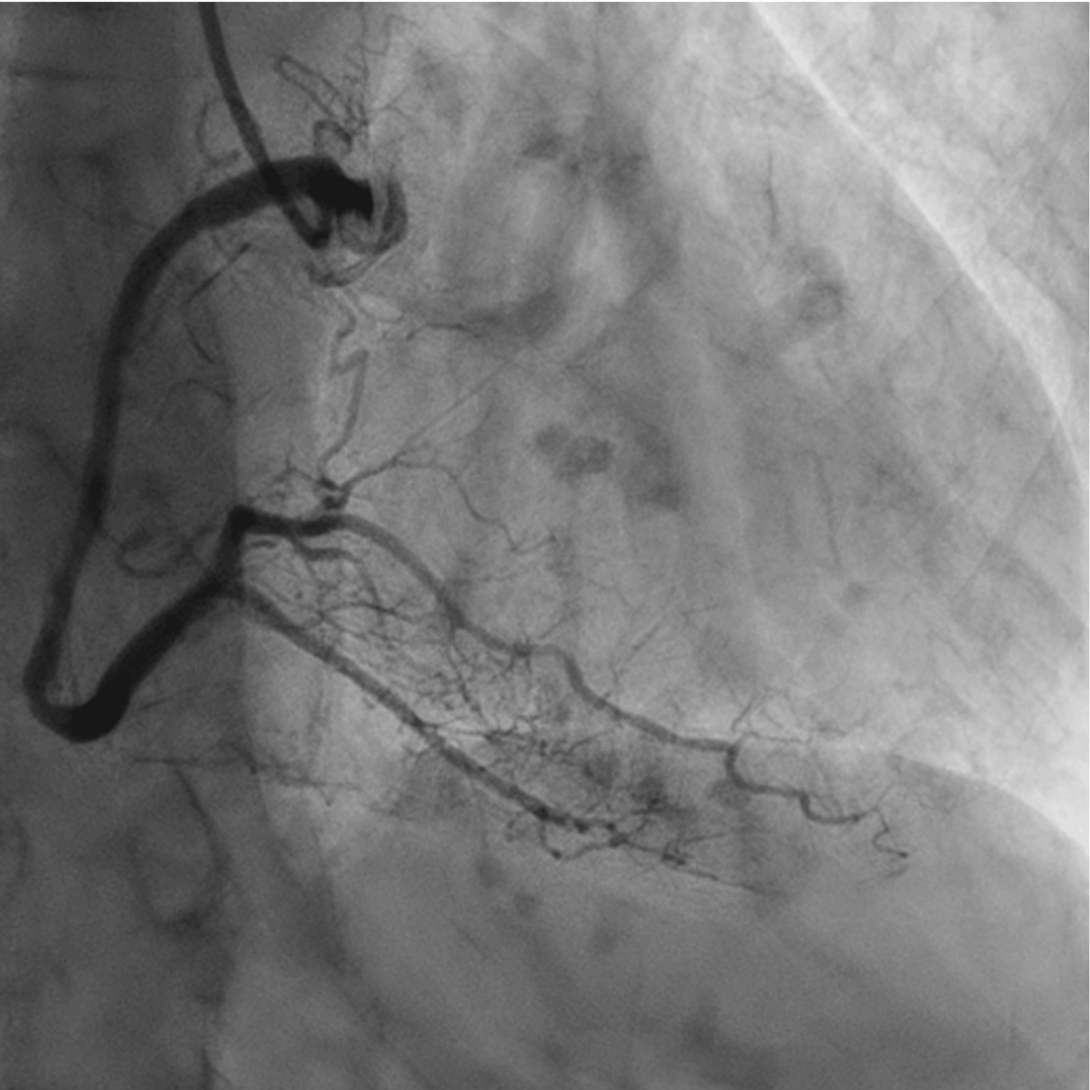
Coronary catheterization of the right coronary artery without evidence of obstruction.

**Figure 6:**
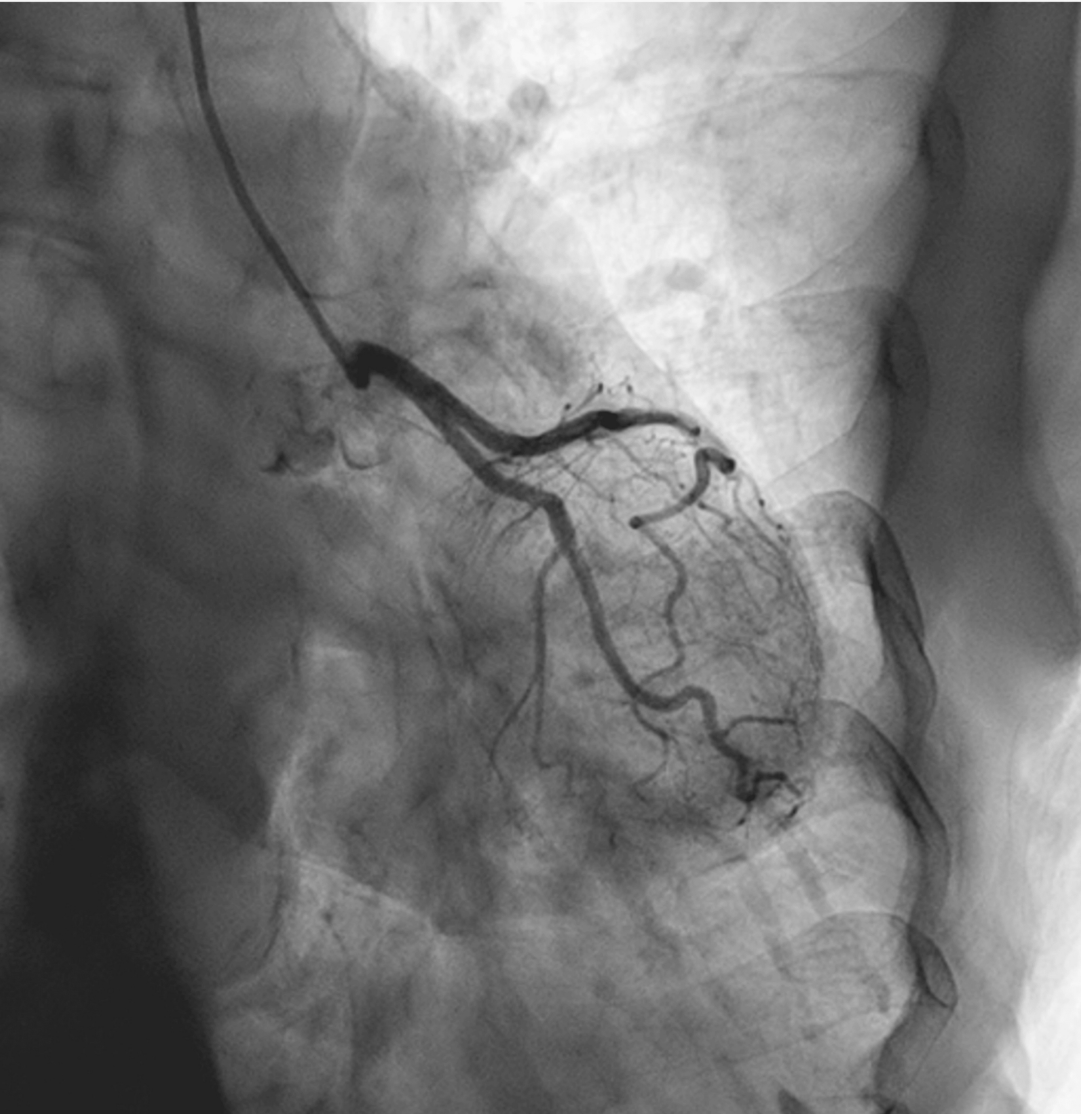
Coronary catheterization of the left circumflex artery without evidence of obstruction.

**Figure 7:**
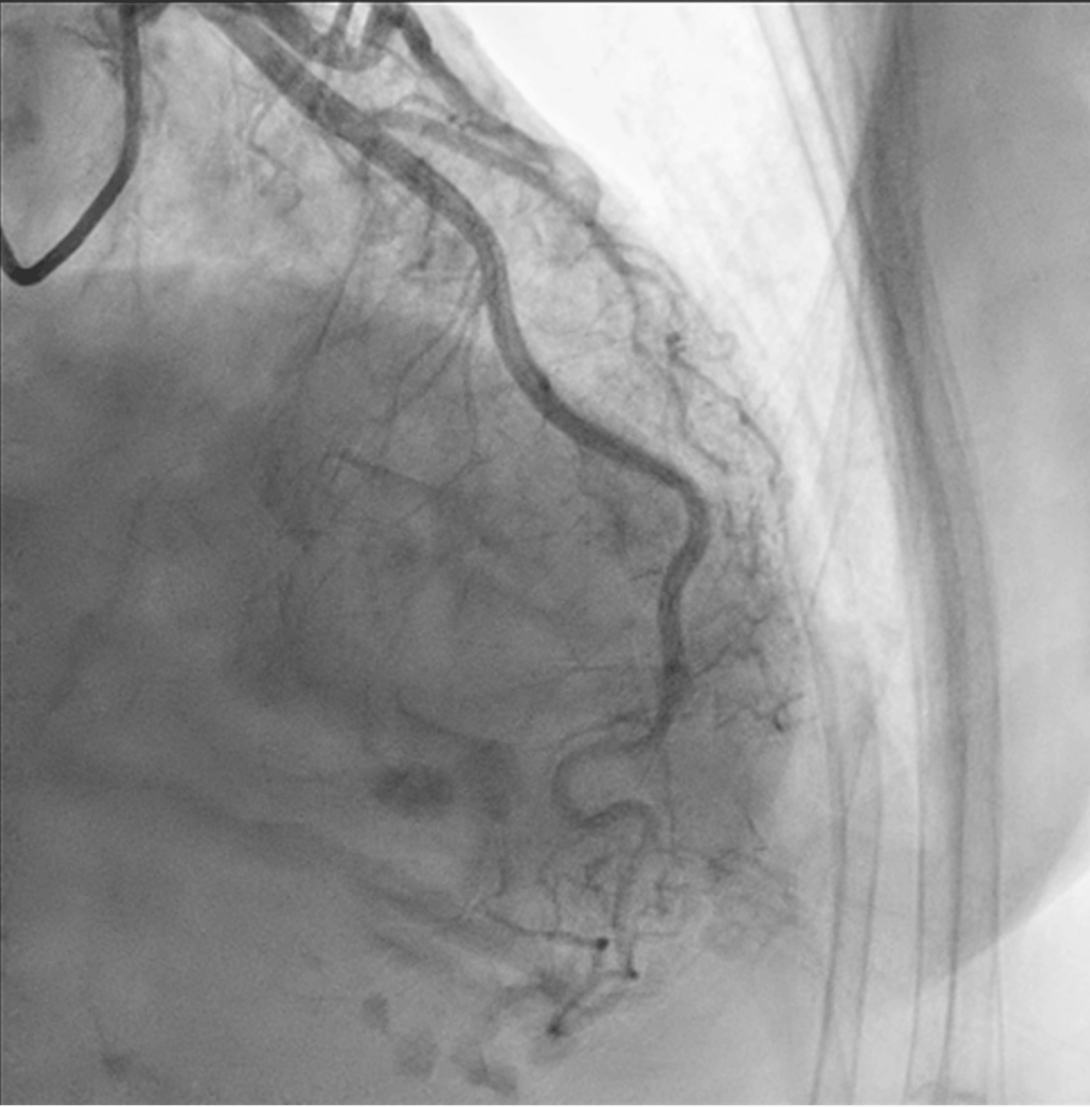
Coronary catheterization of the left anterior descending artery without evidence of obstruction.

Given the results of the initial workup in the emergency department, a transthoracic echocardiogram (TTE) was subsequently performed. It showed mildly reduced left ventricular systolic function with an ejection fraction of 41%, mild left ventricular enlargement, hypokinetic to akinetic wall movement of the basal and middle portions, and hyperdynamic wall movement of the distal portion and apex (Figures 3-4). The recent findings on the updated echocardiogram, with the hypokinetic/akinetic wall movement at the basal segment, were suggestive of the reverse type variant of Takotsubo cardiomyopathy. This was in stark contrast to an echocardiogram performed three weeks prior, which showed a normal ejection fraction of 55% without regional wall motion abnormalities. Furthermore, cardiac coronary catheterization of the main vessels did not reveal any obstruction or coronary artery disease (Figures 5-7).

The patient was stabilized and treated with guideline-directed medical therapy, including metoprolol succinate, atorvastatin, and sacubitril-valsartan. Spironolactone was initiated but held due to hyperkalemia. The patient was discharged with a plan to follow up with outpatient cardiology. At follow-up, the patient reported no complaints of chest pain with exertion or shortness of breath. The patient was instructed to schedule a TTE to assess left ventricular ejection fraction, but this was not completed.

## Discussion

TTC is a clinical condition that closely mimics ACS without any evident coronary obstruction on an angiogram. The exact pathophysiology of TTC is unknown; some common theories of the cause include catecholamine-induced cardiotoxicity, coronary artery spasm, coronary microvascular impairment, and estrogen deficiency [1,5]. The most commonly recognized triggers of rTTC are physical and emotional stressors [1-2,5]. Less commonly recognized triggers include general anesthesia, eating disorders, multiple sclerosis, and exogenous catecholamine use [1,3]. There have also been other cases reporting surgery as a possible trigger of rTTC. For example, there was a recent case report of a 61-year-old man who had a trigger or rTTC following liver transplant surgery [6]. Following transplantation, the patient experienced cardiogenic shock and was discovered to have rTTC. However, other documented surgical cases resulting in rTTC have a typical demographics such as being young and of female gender.

In one such occurrence, a younger female patient experienced rTTC after an exploratory laparotomy. Her medical history was significant for stable multiple sclerosis, which can be a trigger; however, previous cases of rTTC with this risk factor occurred in the absence of surgery [3,7]. Another factor that cannot be overlooked during a surgical procedure is the role of general anesthesia in the precipitation of rTTC. Assessing either alone as a risk factor is extremely difficult. In a case reported by Zhang et al., the patient was hypoxic and was in ventricular fibrillation shortly after undergoing anesthesia [8]. The report determined that anesthesia alone could not be established as the sole inciting event of rTTC. More so, hypoxia, a physiological stressor, could have been the inciting trigger. Other than physiological stressors, emotional stressors can also cause rTTC, as given in Barbaryan et al. [9].

In our case, the elderly woman did have a recent stressful emotional event; however, this occurred after the procedure. As noted in the patient history, she felt tired immediately following surgery, which points to a less likely role of the emotional event as the cause. A case of rTTC has also been reported in a young healthy 27-year-old female without any significant medical history who underwent endoscopic sinus surgery [10]. That patient eventually developed ventricular fibrillation after the procedure and was discovered to have rTTC. There is a probable increased risk of developing rTTC with either anesthesia use or the surgical procedure itself. Differentiating the exact risk factor has been difficult due to the heterogeneity of the procedures. There is also difficulty in isolating general anesthesia use from surgery as they are needed in conjunction with one another. However, there is one other case in the literature that reported rTTC occurrence after a laparoscopic cholecystectomy with a similar decrease in ejection fraction (42% vs 41%) as our patient [11]. The main differences between our patient and the patient referenced in Acar et al. [11] were age and history of cardiac disease. In both cases, there was no noted incidence of hypoxic insult or other anesthetic complications reported. As previously mentioned, there were also no anesthetic complications in the young female patient who underwent endoscopic sinus surgery. The low likelihood of anesthesia being the cause in the endoscopic sinus surgery case, coupled with the similarities between the case of Acar et al. [11] and our case, led us to believe that surgery was a likely risk factor for rTTC. Due to the absence of the aforementioned causes and without any other known risk factors or triggers, the surgery itself can most likely be considered a physiological stressor and probable cause of rTTC. Therefore, it is important to disclose this information during presurgical discussions as part of possible complications as well as being able to recognize rTTC in the postoperative period.

## Conclusions

With the advances made in imaging, clinicians are now able to better diagnose and differentiate between the subtypes of TTC. In the case of our elderly patient presenting with rTTC, imaging demonstrated a unique presentation due to her age demographic, and occurrence following the laparoscopic cholecystectomy procedure. The laparoscopic cholecystectomy procedure was likely the provoking stressor as opposed to the more commonly known risk factors in rTTC. The belief that rTTC occurred after surgery held true due to the convincing evidence that the patient experienced symptoms immediately after the operative period without any reported complications during the procedure. With the stress overdrive that can be associated with surgical operations, having a presurgical discussion with all the known risks involved is important to allow patients to make better-informed decisions about their health. Due to the limited literature on risk factors of rTTC, this case report should be useful for clinicians to better recognize this variant to guide future treatment decisions.
